# Antimicrobial Susceptibility and Genomic Profiles of Multidrug-Resistant *Staphylococcus aureus* from Nasopharynx of Asymptomatic Children in Dhaka, Bangladesh

**DOI:** 10.3390/life14080971

**Published:** 2024-08-02

**Authors:** Sufia Islam, Nishat Nasrin, Nigar Sultana Tithi, Farjana Khatun, Muhammad Asaduzzaman, Anika Fatema Topa, Md Farhad Kabir, Fahim Kabir Monjurul Haque, Mohammad Jubair, Mustafizur Rahman, Christian Lehmann

**Affiliations:** 1 Department of Pharmacy, East West University, Dhaka 1212, Bangladesh; nishat@ewubd.edu (N.N.); ngst@ewubd.edu (N.S.T.); fkh@ewubd.edu (F.K.); anikafatematopa58@gmail.com (A.F.T.); 2 Department of Clinical Pharmacy and Pharmacology, University of Dhaka, Dhaka 1000, Bangladesh; asaduzzaman@du.ac.bd; 3 School of Pharmacy, BRAC University, Dhaka 1212, Bangladesh; 4 Nutrition & Clinical Services Division, International Centre for Diarrhoeal Disease Research, Bangladesh (icddr,b), Dhaka 1212, Bangladesh; farhadkabir@icddrb.org; 5 Department of Mathematics and Natural Sciences, BRAC University, Dhaka 1212, Bangladesh; fahim.haque@bracu.ac.bd; 6 Genome Centre, Infectious Diseases Division, International Centre for Diarrhoeal Disease Research, Bangladesh (icddr,b), Dhaka 1212, Bangladesh; mohammad.jubair@icddrb.org (M.J.); mustafizur@icddrb.org (M.R.); 7 Virology Laboratory, Infectious Diseases Division, International Centre for Diarrhoeal Disease Research, Bangladesh (icddr,b), Dhaka 1212, Bangladesh; 8 Department of Anesthesia, Pain Management and Perioperative Medicine, Faculty of Medicine, Dalhousie University, Halifax, NS B3H 1X5, Canada; chlehmann@mac.com

**Keywords:** nasopharyngeal samples, *S. aureus*, methicillin-resistant *S. aureus*, multidrug-resistant, ST80, antimicrobial resistance genes

## Abstract

Children carrying *Staphylococcus aureus* in their nasopharynx are at a higher risk of contracting systemic infection. Due to lack of sufficient information regarding such carriage, this study was conducted to explore the prevalence, antibiotic susceptibility, and genomic profiles of *S. aureus* isolated from nasopharyngeal samples of 163 randomly selected asymptomatic Bangladeshi children aged from 5–<15 years. Antibiotic susceptibility pattern and genomic analysis of the samples were conducted using standard microbiological methods and genomic tools. The carriage was confirmed in 44 (27%) children who were mostly well nourished without respiratory symptoms in the last 3 months. Higher carriage was observed among the younger age group (5–<10 years) who completed vaccines for pneumonia (*p* = 0.002) and influenza (*p* = 0.004). Among the isolates, 84.1% were multidrug-resistant and 47.5% (n = 40) were methicillin-resistant *S. aureus* (MRSA). All the isolates (100%) were resistant to cefixime with higher resistance to ampicillin (95.5%) and penicillin (90.9%). Among the three investigated isolates, two were ST80 (ID-1 and ID-52) and one was a novel strain (ID-19) with the presence of *aph*-S*tph*, *blaI*, *blaZ*, *dha1*, *fosB*, *lmrS*, *mepA*, *norA*, and *tet*38 genes. The current research demonstrates a high incidence of multidrug-resistant *S. aureus* and reports the first instance of ST80 in asymptomatic children in Bangladesh.

## 1. Introduction

The nasopharynx and anterior nares are considered the major habitats of *S. aureus* [1,2], with nasal transmission observed from 16.8% to 90% of strains [1]. When *S. aureus* is acquired as an asymptomatic nasal carriage, it can gradually turn into a persistent carriage where children are especially susceptible to infection due to the systemic invasion of the pathogen [2]. According to a study conducted by Esposito et al., out of the 497 healthy subjects, 264 (53.1%) were carriers of *S. aureus*, with 195 (39.2%) being nasal carriers. However, the study revealed that only three nasal samples (0.6%) were methicillin-resistant *S. aureus* (MRSA), indicating that the prevalence of MRSA nasal carriage among healthy individuals is relatively low [3]. There are few studies on the prevalence of *S. aureus* in children. Studies in Nepal and Vietnam found *S. aureus* in the upper respiratory tract in 15% and 29.8% of children, respectively [4,5]. Over the years, *S. aureus* has developed resistance against penicillin and methicillin, which were once effective treatments for the bacteria in the mid-20th century [6]. Despite advancements in antibiotic therapy, MRSA still poses a significant risk to global health. Reports from different studies showed that the MRSA isolates were resistant to ciprofloxacin (88.9%) and erythromycin (72.2%) among the identified cases. It was also found that about 20 to 100% of *S. aureus* isolates were susceptible to different antibiotics [7,8]. In China and other Asian countries, the most prevalent hospital acquired-MRSA clones were ST239 and ST5, while community acquired-MRSA clones were diverse such as ST59, ST338, ST30, ST72, and ST8 [9,10]. There were two reports of ST80 MRSA isolates among infected patients in Malaysia [11] and Bangladesh [12]. It is well documented that MRSA-ST80 isolate is considered a multidrug-resistant pathogen due to its resistance to more than three classes of antibiotics [13]. Microbes and human interaction, environmental factors, and host characteristics, including both innate and adaptive immune responses, can predispose to colonization of *S. aureus* and can result in opportunistic and occasionally life-threatening infections that raise morbidity, mortality, and healthcare costs [14,15]. Therefore, it is important to monitor the strains of *S. aureus* found in the nasopharyngeal carriage since they may be the main source of bacterial dispersion in the community and the primary reservoir of respiratory infections. Screening in the early years of life could be worthwhile to obtain a more precise estimate of *S. aureus* circulation. There is a lack of information regarding the prevalence of *S. aureus* strains among the asymptomatic Bangladeshi children. Therefore, this study was designed to explore the nasopharyngeal carriage of S. *aureus* and their resistance pattern against available antimicrobial agents as well as the genomic profiles of selected isolates among children of different age groups.

## 2. Materials and Methods

### 2.1. Study Population

This study was carried out between April 2022 and January 2023. Nasopharyngeal samples were collected from 163 asymptomatic, and hence, apparently healthy school children aged between 5 and <15 years from 14 randomly selected schools situated in Dhaka, Bangladesh. Among them, 75 children were from 5–<10 years of age, whereas 88 children were aged from 10–<15 years. Children who suffered from infection or in an unhealthy physical state for swab collection or under antibacterial therapy 15 days before sample collection were excluded from the study.

### 2.2. Sociodemographic Characteristics and Nutritional Status

Sociodemographic characteristics, including socioeconomic status, family size, number of siblings, housing condition, education level, and employment status of parents, were assessed in this present study. Nutritional assessment (height for age Z scores, BMI for age Z scores) of each participant was determined [16,17]. Other parameters, including vaccination status, episodes of respiratory symptoms, and antibiotic consumption history, were also evaluated.

### 2.3. Nasopharyngeal Specimen Collection and Identification of Bacterial Isolates

Nasopharyngeal specimens were collected using sterile swabs following standard procedure and then cultured onto mannitol salt agar (MSA) media (HiMedia, Mumbai, India). Bacterial isolates were identified based on their colony morphology and biochemical tests according to standard microbiological methods [18,19].

### 2.4. Antibacterial Susceptibility Test

The disk diffusion method was used for the antibiotic susceptibility testing according to the Clinical and Laboratory Standards Institute (CLSI) guidelines [20] using *S. aureus* (ATCC 25923) strain as the control. The isolates were screened for susceptibility to penicillin (10 units), ampicillin (25 µg), amoxyclav (30 µg), sulfamethoxazole-trimethoprim or cotrimoxazole (25 µg), cefixime (5 µg), azithromycin (15 µg), erythromycin (15 µg), ceftriaxone (30 µg), chloramphenicol (30 µg), ciprofloxacin (5 µg), levofloxacin (5 µg), gentamicin (10 µg), tobramycin (10 µg), imipenem (10 µg), and meropenem (10 µg) (HiMedia, India). From 44 positive strains, 40 isolates were further analyzed for confirmation using VITEK^®^ 2 system (bioMérieux, Durham, NC, USA) [21]. Isolates resistant to 30 µg cefoxitin were classified as MRSA [22].

### 2.5. Whole Genome Analysis

Whole genome analysis was performed on three representative isolates: two were MRSA, and one was methicillin-sensitive *S. aureus* (MSSA). The isolates for genomic analysis were selected based on their antibiotic sensitivity pattern. The MRSA isolates exhibited resistance to most of the therapeutic class of antibiotics, while the MSSA was susceptible. Both MRSA and MSSA isolates were investigated to obtain valuable insights for comparing their resistant phenotypes and genomic profiles.

DNA extraction was performed using the Qiagen DNeasy Blood & Tissue Kit (250) with modification, and the quality of the extracted DNA was evaluated for suitability for subsequent whole genome sequencing (WGS) using both Nanodrop and Qubit measurements. DNA purification was carried out by a modified version of the CDC PULSENET protocol [12]. For library preparation, the Illumina DNA Prep Reagent Kit and an automated liquid handler (epMotion 5075) were utilized. The prepared DNA libraries were subjected to sequencing using the Illumina NextSeq550 platform. Sequencing was performed with paired-end 2 × 150 bp reads, yielding comprehensive genetic information. The Phoenix pipeline analyzed raw paired-end reads from the NextSeq550 platform (https://github.com/CDCgov/phoenix) (accessed on 25 February 2024). Species identification was carried out to confirm the organism from which the genomic data were derived. Subsequently, the data underwent a trimming process to remove any adapters and low-quality sequences, employing tools such as BBDuk (https://github.com/BioInfoTools/BBMap) (accessed on 25 February 2024) and Fastp (https://github.com/OpenGene/fastp) (accessed on 25 February 2024). The high-quality, trimmed reads were assembled using the SPAdes [23] algorithm. Followed by sequence typing, antimicrobial resistance (AR) genes were identified utilizing the tools available in AR Gene Curated Databases, such as Resfinder, NCBI, and ARG-ANNOT. 

For sequence type determination, SISTR (https://github.com/katholt/srst2) (accessed on 25 February 2024) and MLST (https://github.com/tseemann/mlst) (accessed on 25 February 2024) were employed with a custom MLST database for additional specificity. The scaffolds were annotated using the Prokka (https://github.com/tseemann/prokka) (accessed on 25 February 2024) annotation tool and AMRFinder (https://ftp.ncbi.nlm.nih.gov/pathogen/Antimicrobial_resistance/AMRFinderPlus/database/) (accessed on 25 February 2024) to identify antimicrobial resistance genes. The annotated genomic data were subjected to comparative genomic analysis by using GAMMA (https://github.com/rastanton/GAMMA) (accessed on 25 February 2024) and Kraken2 (https://github.com/DerrickWood/kraken2/wiki/Manual) (accessed on 25 February 2024). Plasmid presence was investigated using PlasmidFinder (https://pubmed.ncbi.nlm.nih.gov/31584170/) (accessed on 25 February 2024), and the genomic data were also scanned for hypervirulence genes to identify any potential virulence factors. The sequence data processed during the current study were compiled, prepared according to the guideline, and submitted to the NCBI (BioProject accession no. PRJNA1109619).

### 2.6. Statistical Analysis

The data were analyzed using Stata software (V.15, StataCorp, College Station, TX, USA). Descriptive statistics, including frequency and proportions, were used to summarize categorical variables, while mean and standard deviation (SD) or median with interquartile range (IQR) were used for quantitative variables. The association between categorical variables was assessed using the Chi-square test or Fisher’s exact test, depending on the sample size. For quantitative variables, the association was determined using either the *t*-test or Mann–Whitney U test, depending on the data distribution. A *p*-value of less than 0.05 was considered as a statistically significant value.

### 2.7. Ethics Statement

This study was approved by the East West University Research Ethical Committee (Ethical Clearance no. EWUCRT-REC-3/2019) and was conducted in compliance with the Declaration of Helsinki. Informed written consent was obtained from the parents/guardians of all participants before conducting the study.

## 3. Results

### 3.1. S. aureus Carriage, Patient Demography, Vaccination and Antibiotic Use Profiles

From a total of 163 healthy children, 66 (40.5%) were male and 97 (59.5%) were female. Nasopharyngeal swab samples of 44 (27%) participants were confirmed for *S. aureus* carriage with an equal distribution between the two age groups (5–<10 years and 10–<15 years). *S. aureus* carriage was significantly higher in male compared to the female (*p* = 0.01). The monthly family income, education, and occupation of the parents indicated that they were from lower middle-class families [24,25]. Table 1 shows that the majority of the *S. aureus* carriers were well nourished children (86.4%) in terms of both BMIZ values and stunting. Among *S. aureus* carriers from the 5–<10 years age group, about 95.2% of children have completed their EPI vaccination (*p* < 0.001), 90.5% received the Hib vaccine (*p* = 0.004), and 85.7% received the PCV vaccine (*p* = 0.002), which were significantly higher than children from the 10–<15 years age group. Moreover, a larger proportion of younger carriers had respiratory episodes within the last 3 months of the study than the older ones (*p* = 0.031) (Table 1).

### 3.2. Antibiotic Resistance Profile

Resistance profiles of the isolated bacteria against the 15 antibiotics are presented in Table 2. All *S. aureus* isolates were resistant to cefixime, but none showed resistance against gentamicin. Higher resistance was observed against ampicillin (95.5%), penicillin (90.9%), erythromycin (68.2%), and azithromycin (63.6%). Isolates from 5–<10-year-old children showed slightly higher resistance to ampicillin and penicillin compared to older children (100% vs. 90.9% and 95.5% vs. 86.4%, respectively), whereas resistance against azithromycin was slightly higher among 10–<15-year-old children. *S. aureus* isolates showed higher sensitivity against tobramycin, cotrimoxazole, and chloramphenicol. 

**Figure 1:**
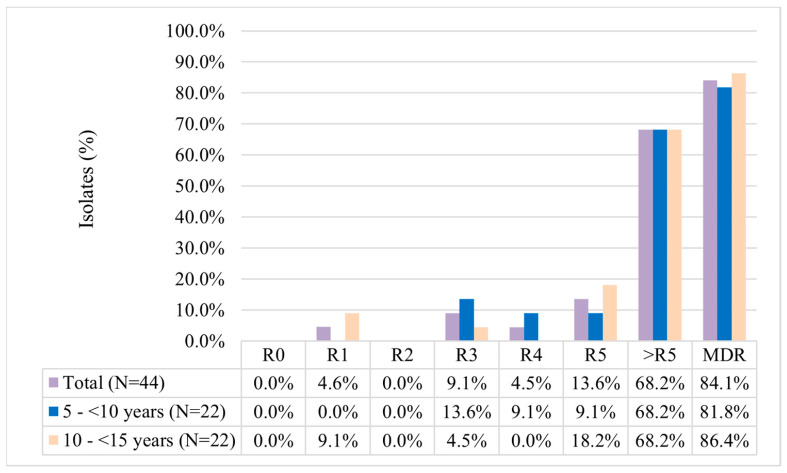
Antibiotic resistance pattern of *S. aureus* among different age groups. R0: no antibiotic resistance; R1 to R5: resistance to 1 antibiotic to 5 antibiotics; and >R5: resistance to 6 to 14 antibiotics; MDR: resistance to 3 or more classes of antibiotics. Abbreviation: MDR, multidrug-resistant.

Multidrug resistance was observed in 84.1% of *S. aureus* isolates (Figure 1), with a higher proportion among 10–<15-year-old children (86.4%). About 68.2% of the isolates showed resistance against six or more antibiotics. About 47.5% (n = 40) of *S. aureus* isolates showed resistance to cefoxitin, and hence, considered as MRSA. 

Common resistance phenotypes among the multidrug-resistant isolates are shown in Table 3.

### 3.3. Whole Genome Analysis and Phenotype–Genotype Correlation

The genome quality and contents of the organisms specified were satisfactory, as revealed by the Phoenix output. Overall, the quality of the isolates was also satisfactory, indicating a higher coverage depth of the genomes, ranging from 70× to 130×. The assembly ratio of the genome, which is the ratio between the total number of bases in the sample assembly compared to the expected genome size, was less than 2.58. The number of scaffolds found was also less than 50, indicating a smaller gap and much better genome quality. Additionally, the GC content (32%) was also very satisfactory. The taxonomic identification process of Phoenix is carried out by three tools, namely, MLST, ANI (Average Nucleotide Identity), and Kmerfinder. However, the MLST result shows sequence type of ST 80 for ID-1 and ID-52 and Novel allele type is indicated for ID-19. With the help of the Kraken tool, only one genus (confirmed as *S. aureus*) has been found above the 25% threshold, which indicates no contamination of other species.

Genotype analysis of three isolates of *S. aureus* (ID-1, ID-19, and ID-52) revealed that they possessed six antimicrobial resistance (AMR) genes—*aph*-S*tph*, *dha1*, *norA*, *tet*38, *mepA*, and *lmrs*—with corresponding resistance to aminoglycosides, phenicols (chloramphenicol), quinolones, tetracyclines (tetracycline and tigecycline), and lincosamides, respectively (Table 4). The ID-1 and ID-52 isolates also contained beta-lactam resistance genes *mecA* (methicillin), *blaI* and *blaZ* (penicillin), and macrolide and lincosamide resistance gene *ermC*. Moreover, these two isolates showed resistance to fluoroquinolone (ciprofloxacin and levofloxacin) due to the presence of two point mutations, one in *gyrA*_S84L and the other in *par*C_S80F, in the quinolone-resistance determining regions (QRDRs) of isolates. They were also characterized by the presence of four different plasmid replicons—*rep7c*, *rep5a* (pN315), *rep10* (pDLK1), and *rep16* (pSaa6159). The isolate ID-19, however, exclusively harbored the *fosB* gene that is known to confer resistance against fosfomycin (Table 4). Findings of the whole genome sequencing, which confirmed ID-1 and ID-52 as MRSA and ID-19 as MSSA corroborate with phenotypic results based on cefoxitin sensitivity as obtained from the VITEK^®^ 2 data.

## 4. Discussion

Asymptomatic colonization of *S. aureus* increases the risk of invasive infections. To our knowledge, this is the very first report from Bangladesh on the nasopharyngeal carriage of *S. aureus* and their resistance pattern in healthy, asymptomatic children.

In the current study, about 27% of nasopharyngeal samples tested positive for *S. aureus*, with 84.1% showing MDR phenotype. Similar to our study, Awulachew et al. reported a global nasopharyngeal carriage of *S. aureus* (25%) in children between the ages of 6 and 15 [26]. According to a cross-sectional investigation, 10.3% of all samples (n = 234), 24 positive cultures contained *S. aureus* in children aged ≤10 years, with a higher carriage rate of 14.3% between 8 and 10 years [27]. In our study, *S. aureus* carriage was significantly higher in male children than in females. This difference may be attributed to the variation in sex chromosomes. Females possess two X chromosomes, each enriched with 1100 genes and harboring several immune function-regulating genes. On the contrary, males having single X chromosomes make them more vulnerable to infection. Additionally, lifestyle factors, exposure to risk factors, and healthcare-seeking behaviors may also contribute to differentiating the carriage rate of *S. aureus* between male and female children [28]. Our research also revealed that *S. aureus* carriage is higher among the children who received PCV and Hib vaccines. The introduction of PCV-13 might have contributed to the rise in *S. aureus* prevalence in the nasopharynx [29].

In a sub-group analysis of 40 multidrug-resistant isolates of *S. aureus*, we found that 47.5% of the isolates were MRSA. Similar results were seen in other studies where the subjects were MRSA carriers [30,31]; however, the carriage rate was lower in those studies than ours. We found 100% cefixime-resistant *S. aureus* in this present study, which, according to our knowledge, has not been reported to date. Different studies demonstrated cefixime resistance in patients against *S. aureus* isolates in Bangladesh. One study demonstrated 80% and 85.2% resistance of the isolates to cefixime from urine samples in males and females, respectively [32]. 

According to various studies, *S. aureus* resistance to erythromycin in school children ranged from 20 to 46% [4,33]. A study from Bangladesh also demonstrated that less than 40% of isolates were sensitive to erythromycin and azithromycin [7]. In our investigation, however, *S. aureus* exhibited comparatively higher macrolide resistance (63.3% for azithromycin and 68.2% for erythromycin). 

As revealed from epidemiological data, MRSA-ST80 is prevalent geographically in case of hospital and community-acquired infections. It has been identified in Europe (23 countries), Middle East (10 countries), North Africa (3 countries), and Asia (2 countries). In Asia, ST-80 isolates were detected in Malaysia and Bangladesh. In Malaysia, only one strain (1/154 MRSA) was identified from the nasal carriage of a dialyzed patient [11]. In Bangladesh, 3.8% (5/132) of MRSA strains have been identified as ST80 [12], which harbored both *pvl* and *mecA* genes. In contrast, two of our samples were found to be *pvl*-negative ST-80 harboring *mecA* gene. These two MRSA-ST80 isolates were multidrug-resistant and characterized by the presence of genes responsible for beta-lactam (*blaI*, *blaZ*, *mecA*), macrolide (*ermC*), aminoglycoside (*aph*-S*tph*), lincomycin (*lmrS*), tetracyclines (*mepA* and *tet*38), and fluoroquinolone (*norA*, *gyrA_s84L* and *parC_s80F*) resistance. Another isolate, ID-19, appeared as a novel allele which lacks *blaI*, *blaZ* and *ermC* genes but contained *fosB* gene and *rep7c* plasmid replicon.

Research has shown that the *aac(6′)-Ie-aph (2″)* and *aph(3′)-IIIa* genes demonstrate a vital role in developing resistance to aminoglycoside antibiotics [34,35]. In this study, these three isolates were found to possess the aminoglycoside phosphotransferase (APH) gene, *aph*-S*tph*. However, they still showed sensitivity to aminoglycoside antibiotics (gentamicin and tobramycin). 

All three investigated isolates harbored both *tet*38 and *mepA* genes, which are linked to the antibiotic efflux mechanism; however, isolates ID-1 and ID-52 were found to be sensitive to tetracycline and tigecycline, respectively, in phenotype analysis. A study carried out by Zeng et. al., reported that the active *tet*K efflux pump was primarily responsible for tetracycline resistance [36]. This study also observed mutation of the *mepA* gene and premature termination of the respective efflux protein in tetracycline-sensitive isolates. Moreover, low expression of *tet*38 and *mepA* genes was also responsible for tetracycline sensitivity [36]. The *mepA* protein pump is regulated by *mep*R, which is a part of *mep*RAB cluster [37]. This explains the sensitivity profile of investigated isolates for tetracyclines and other antibiotics (aminoglycosides and chloramphenicol) in the present study. 

Additionally, these isolates possess an MDR gene, *lmrs*, which confers resistance to aminoglycoside, macrolide, phenicol, diaminopyrimidine (trimethoprim), and oxazolidinone antibiotics [37]. The expression of this gene might have been relatively higher in isolate ID-1, therefore showing intermediate resistance to cotrimoxazole (trimethoprim and sulfamethoxazole). 

Plasmids bear mobile genetic elements (MGEs) and are the major means of horizontal transfer of resistance determinants and virulence factors [38]. Isolates ID-1 and ID-52 were characterized by the presence of four different plasmid replicons—*rep7c*, *rep5a* (pN315), *rep10* (pDLK1), and *rep16* (pSaa6159). The *rep5a* was reported as a common plasmid replicon in Malaysian MRSA isolates [29], which is similar to our findings. Moreover, the acquisition of *rep10* and *rep16* has been found to be responsible for macrolide and lincosamide, and beta-lactam antibiotic resistance, as *rep10* and *rep16* plasmid replicons harbored *ermC* and *blaZ* genes, respectively, and thus have been implicated in the transmission of genetic information among species [29,39]. In Central China, pork samples were reported to contain *S. aureus* harboring *rep10* and *rep16* [40]. The presence of these resistant genes might be useful in understanding the spread of resistant pathogens in Bangladesh, as found in other Asian countries. Therefore, understanding the distribution and function of MGEs and plasmids in *S. aureus* is crucial for combating antibiotic resistance. This understanding can aid in monitoring MGEs to track the spread of resistance, while genomic analyses provide a comprehensive view of resistance scenarios. Additionally, studying the mechanisms of MGEs and plasmids enables researchers to predict resistance trends, design targeted interventions, and develop strategies to prevent gene transfer. Practical strategies may include infection control, antibiotic stewardship, and research into novel treatments. Overall, this understanding is essential for devising effective measures to manage and mitigate antibiotic resistance. 

A unique aspect of this study is detecting antibiotic-resistant microorganisms in nasopharyngeal samples obtained from asymptomatic school children. It includes a wide range of variables to better understand the potential connections to antimicrobial resistance in apparently healthy children, which is a gap in current research. Additionally, previous studies have not thoroughly examined various schools in Dhaka city in a single publication to comprehend patterns of antimicrobial resistance. By involving children from 14 schools in Dhaka city, our study underscores its scientific robustness. While we acknowledge that the sample population may not perfectly represent the entire nation, we are confident in our findings regarding the nasopharyngeal carriage of *S. aureus*. However, further studies are needed with data collected from various districts of Bangladesh to gain a more comprehensive understanding of the prevailing situation. 

## 5. Conclusions

In our study, we detected the presence of multidrug-resistant ST80 MRSA isolates, which is, to our best knowledge, the first report from Bangladesh among asymptomatic children. *S. aureus* is prevalent in nasopharyngeal carriage among school children in Dhaka, and most of these isolates were multidrug-resistant. MRSA was also detected in these samples. One of the reasons for multidrug resistance may be the indiscriminate use of antibiotics among children. It is essential for the nationwide antimicrobial resistance (AMR) surveillance program to consistently monitor antimicrobial resistance to *S. aureus* in the population, including children. Promoting the responsible use of antibiotics across the nation, implementing routine screening for AMR, and ensuring that policymakers take necessary actions to control the issue are crucial strategies. Public awareness of antibiotic usage restrictions should also be implemented.

The present study on the antimicrobial susceptibility and genomic profile of *Staphylococcus aureus* nasal carriage in children in Dhaka, Bangladesh, provides valuable insights into understanding the prevalence of this bacterium and its susceptibility to antimicrobial agents. Continuing a thorough longitudinal study with a substantial sample size is crucial to validate the findings. This approach will allow us to effectively integrate our research into comprehending the modes of contamination and transmission of bacteria, especially in identifying *S. aureus* in siblings and school groups. Moreover, exploring the prevalence and diversity of MGEs in larger and more diverse *S. aureus* strains and conducting functional studies on the identified plasmid replicons and their encoded resistance genes will provide deeper insights into their roles in resistance and virulence. The outcomes can then be utilized to formulate effective prevention and treatment strategies to tackle the issue. 

## Figures and Tables

**Table 1:** Age group-wise distribution of *S. aureus* carriers.

Variables		Total (n = 44)	5–<10 Years 22 (50)	10–<15 Years 22 (50)	*p*-Value
Sex	Male	25 (56.8)	12 (54.5)	13 (59.1)	1.000 ^a^
	Female	19 (43.2)	10 (45.5)	9 (40.9)	
Children BMI		16.3 ± 3.2	16.08 ± 3.8	16.56 ± 2.45	0.628 ^c^
Family size		5 (4, 6)	5 (4, 5)	6 (5, 10)	0.008 ^d^
No. of siblings		3 (1, 3)	3 (1, 3)	2 (1, 3)	0.942 ^d^
Persons living per room		5 (4, 6)	5 (4, 5)	6 (4, 6)	0.064 ^d^
Monthly family income (1000 BDT)		12 (10, 15)	12 (10, 15)	10 (8, 13)	0.111 ^d^
Nutritional status					
BMIZ (BMI for age)	Well-nourished	38 (86.4)	19 (86.4)	19 (86.4)	1.000 ^a^
	Malnourished	6 (13.6)	3 (13.6)	3 (13.6)	
Stunting (height for age)	Not stunted	38 (86.4)	21 (95.5)	17 (77.3)	0.185 ^a^
	Stunted	6 (13.6)	1 (4.5)	5 (22.7)	
EPI vaccination status (n = 145)	Complete	28 (68.3)	20 (95.2)	8 (40)	<0.001 ^a^
	Incomplete	13 (31.7)	1 (4.8)	12 (60)	
Hib vaccination status (n = 145)	Complete	29 (70.7)	19 (90.5)	10 (50)	0.004 ^b^
	Incomplete	12 (29.3)	2 (9.5)	10 (50)	
PCV vaccination status (n = 145)	Complete	26 (63.4)	18 (85.7)	8 (40)	0.002 ^b^
	Incomplete	15 (36.6)	3 (14.3)	12 (60)	
Respiratory episode in last 3 months (n = 145)	No episode	15 (36.6)	11 (52.4)	4 (20)	0.031 ^b^
	One or more	26 (63.4)	10 (47.6)	16 (80)	
Antibiotic used in last 6 months (n = 145)	Yes	5 (12.2)	3 (14.3)	2 (10)	1.000 ^a^
	No	36 (87.8)	18 (85.7)	18 (90)	

Data are presented as number, n (%). BMI is represented as mean ± SD, whereas family size, number of siblings, persons living per room, and family income are presented as median with interquartile range (IQR). ^a^ Fisher’s exact test; ^b^ Chi-square test; ^c^ Student’s *t*-test; ^d^ Mann–Whitney U test. Abbreviations: BMI, body mass index; BMIZ, body mass index for age; EPI, expanded program on immunization; Hib, *Haemophilus influenzae* type b; PCV, pneumococcal conjugate vaccine.

**Table 2:** Age group-wise distribution of antibiotic-resistant *S. aureus* and MRSA.

Antibiotic Class	Name of Antibiotic	*Staphylococcus aureus*				MRSA			
		Total (n = 44)	5–<10 Years (n = 22)	10–<15 Years (n = 22)	*p*-Value	Total (n = 19)	5–<10 Years (n = 10)	10–<15 Years (n = 9)	*p*-Value
Penicillin	Penicillin	40 (90.9)	21 (95.5)	19 (86.4)	0.607 ^a^	18 (94.7)	9 (90)	9 (100)	1.000 ^a^
	Ampicillin	42 (95.5)	22 (100)	20 (90.9)	0.488 ^a^	19 (100)	10 (100)	9 (100)	-
	Amoxyclav	27 (61.4)	12 (54.5)	15 (68.2)	0.353 ^b^	13 (68.4)	5 (50)	8 (88.9)	0.141 ^a^
Cephalosporin	Cefixime	44 (100)	22 (100)	22 (100)	-	19 (100)	10 (100)	9 (100)	-
	Ceftriaxone	19 (43.2)	7 (31.8)	12 (54.5)	0.128 ^b^	13 (68.4)	4 (40)	9 (100)	0.011 ^a^
Carbapenem	Meropenem	21 (47.7)	9 (40.9)	12 (54.5)	0.365 ^b^	12(63.2)	4 (40)	8 (88.9)	0.057 ^a^
	Imipenem	13 (29.5)	5 (22.7)	8 (36.4)	0.322 ^b^	8 (42.1)	3 (30)	5 (55.6)	0.370 ^a^
Macrolide	Azithromycin	28 (63.6)	13 (59.1)	15 (68.2)	0.531 ^b^	12 (63.2)	5 (50)	7 (77.8)	0.350 ^a^
	Erythromycin	30 (68.2)	15 (68.2)	15 (68.2)	1.000 ^b^	12 (63.2)	5 (50)	7 (77.8)	0.350 ^a^
Aminoglycoside	Gentamicin	0 (0)	0 (0)	0 (0)	-	0 (0)	0 (0)	0 (0)	-
	Tobramycin	1 (2.3)	1 (4.5)	0 (0)	1.000 ^a^	1 (5.3)	1 (10)	0 (0)	1.000 ^a^
Phenicol	Chloramphenicol	4 (9.1)	2 (9.1)	2 (9.1)	1.000 ^a^	2 (10.5)	2 (20)	0 (0)	0.474 ^a^
Quinolone	Ciprofloxacin	20 (45.5)	9 (40.9)	11 (50)	0.545 ^b^	11 (57.9)	5 (50)	6 (66.7)	0.650 ^a^
	Levofloxacin	21 (47.7)	10 (45.5)	11 (50)	0.763 ^b^	10 (52.6)	5 (50)	5 (55.6)	1.000 ^a^
Sulfa drug	Cotrimoxazole	3 (6.8)	1 (4.5)	2 (9.1)	1.000 ^a^	2 (10.5)	1 (10)	1 (11.1)	1.000 ^a^

Data are presented as number, n (%). ^a^ Fisher’s exact test; ^b^ Chi-square test. Abbreviations: MRSA, methicillin-resistant *Staphylococcus aureus*.

**Table 3:** Multidrug-resistant phenotypes of *S. aureus* (n = 37).

Phenotypes	No. (%)
CFM-PEN-AMP-AMC-CTR-----	17 (45.9)
CFM-PEN-AMP-AMC-CIP----	6 (16.2)
CFM-PEN-AMP-CTR-CIP-LEV---	2 (5.4)
CFM-PEN-AMP-AMC-AZM-ERY--	2 (5.4)
CFM-PEN-AMP-LEV-AZM--	3 (8.1)
CFM-AMP-CIP-LEV-AZM-ERY	1 (2.7)
CFM-PEN-AMP-CIP-LEV	1 (2.7)
CFM-PEN-AMP-AZM-ERY	2 (5.4)
CFM-AMP-IPM-LEV-CHL	1 (2.7)
CFM-PEN-AMP-AMC-ERY	1 (2.7)
CFM-PEN-AMP-ERY	1 (2.7)

Abbreviations: CFM, cefixime; PEN, penicillin; AMP, ampicillin; AMC, amoxyclav; CTR, ceftriaxone; CIP, ciprofloxacin; LEV, levofloxacin; AZM, azithromycin; ERY, erythromycin; IPM, imipenem; CHL, chloramphenicol.

**Table 4:** Antimicrobial-resistant phenotypes and genotypes of selected samples of *S. aureus*.

Antibiotics	AMR Profiles			Detected AMR Genes			Detected Plasmids		
	ID-1	ID-19	ID-52	ID-1	ID-19	ID-52	ID-1	ID-19	ID-52
**Beta-lactams**				*blaI* *blaZ* *mecA*		*blaI* *blaZ* *mecA*	*rep7c* (MSSA476) *rep5a* (pN315) *rep16* (pSaa6159) *rep10* (pDLK1)	rep7c (MSSA476)	*rep7c* (MSSA476) *rep5a* (pN315) *rep16* (pSaa6159) *rep10* (pDLK1)
Penicillin	R	S	R						
Ampicillin	R	S	R						
Amoxyclav	R	S	R						
Ceftriaxone	R	S	R						
Cefixime	R	R	R						
Meropenem	R	S	R						
Imipenem	R	S	S						
**Aminoglycosides**				*aph*-S*tph*	*aph*-S*tph*	*aph*-S*tph*			
Gentamycin	S	S	S						
Tobramycin	I	S	S						
**Macrolides**				*ermC*		*ermC*			
Azithromycin	R	S	R						
Erythromycin	R	S	R						
**Lincosamides**				*ermC* *lmrS*	*lmrS*	*ermC* *lmrS*			
Clindamycin	R	-	R						
**Phenicols**									
Chloramphenicol	R	S	S	*dha1*	*dha1*	*dha1*			
**Quinolones**				*norA* *gyrA_S84L* *parC_S80F*	*norA*	*norA* *gyrA_S84L* *parC_S80F*			
Ciprofloxacin	R	S	R						
Levofloxacin	R	S	R						
**Tetracyclines**									
Tetracycline	S	-	S	*tet*38	*tet*38	*tet*38			
Tigecycline	S	-	S	*mepA*	*mepA*	*mepA*			
**Sulphonamides**									
Cotrimoxazole	I	S	S						
**Phosphonic**									
Fosfomycin	-	-	-		*fosB-Saur*				

R: resistant; S: sensitive; I: intermediate resistance; ‘-’: not determined. Abbreviations: AMR, antimicrobial resistance; MSSA, methicillin-sensitive *Staphylococcus aureus*.

## Data Availability

The original contributions presented in the study are included in the article/Supplementary Materials, further inquiries can be directed to the corresponding author. Genome sequence data submitted to the NCBI portal under the BioProject Accession No. PRJNA1109619, will be publicly available after the release date (31 December 2024).

## Supplementary Materials

The following supporting information can be downloaded at: https://www.mdpi.com/article/10.3390/life14080971/s1, Table S1: Genome sequencing and gene-related information of selected *S. aureus* isolates.
